# Relationship between uncoupling protein 1 (UCP1) levels and psoriasis

**DOI:** 10.55730/1300-0144.5960

**Published:** 2024-12-30

**Authors:** Fikret AKYÜREK, Fatma TUNCEZ AKYÜREK, Fatma ŞENGÜL BAĞ

**Affiliations:** 1 Department of Medical Biochemistry, Faculty of Medicine, Selçuk University, Konya, Turkiye; 2 Department of Dermatology, Faculty of Medicine, Selçuk University, Konya, Turkiye; 3 Department of Biochemistry, Faculty of Pharmacy, Adıyaman University, Adıyaman, Turkiye

**Keywords:** Psoriasis, uncoupling protein 1, cardiovascular diseases, high-sensitivity C-reactive protein

## Abstract

**Background/aim:**

Psoriasis is a common chronic autoimmune skin disease. Comorbidities increase the mortality risk of the disease. The aim of this study was to investigate the changes in uncoupling protein 1 (UCP1) level in psoriasis patients and evaluate its possible role in the pathogenesis of the disease, focusing on disease severity (Psoriasis Area and Severity Index), dyslipidemia, inflammation, and cardiovascular risk.

**Materials and methods:**

This study included 30 psoriasis patients and 30 healthy individuals as a control group. Serum UCP1 was measured using an ELISA test kit. The laboratory results of psoriasis patients and healthy controls were compared.

**Results:**

UCP1 level was a significant candidate marker for the prediction of psoriatic disease (AUC: 0.708, 95% CI: 0.577–0.819, p = 0.002) with sensitivity of 66.67%, specificity of 76.67%, negative predictive value of 69.7%, and positive predictive value of 74.1%. Simple logistic regression analysis showed that an individual with a UCP1 value below 7.561 had a 73% lower probability (OR: 0.27, 95% CI: 0.08–0.94, p = 0.039) of developing psoriasis than an individual with a UCP1 value above 7.561. Among the biochemical parameters, the high-sensitivity C-reactive protein and triglyceride levels of the patients were significantly higher compared to those of the healthy controls while their high-density lipoprotein levels were lower.

**Conclusion:**

According to the sensitivity (66.67%) and specificity (76.67%) of UCP1, it may be a valuable candidate marker in the diagnosis of psoriasis patients in symptomatic and asymptomatic phases. Further work is needed to substantiate these findings.

## 1. Introduction

Psoriasis is an autoimmune/immune-mediated inflammatory skin disease that is primarily characterized by plaque formation and often occurs on the extensor surfaces [1]. Its worldwide incidence in adults varies between 0.51% and 11.43%, while in children it is between 0% and 1.37% [2]. The histopathological changes associated with the disease and the changes caused by chronic inflammation contribute to the comorbidities. Comorbidities associated with psoriasis include metabolic syndrome, autoimmune diseases, obesity, cardiovascular disease, hypertension, diabetes, and sleep apnea [3–5].

The Psoriasis Area and Severity Index (PASI) is a preferred tool for assessing disease severity, but its use in nonplaque forms of psoriasis is limited. As it is subjective and time-consuming and it does not provide sufficient data for the assessment of comorbidities, more supportive and practical parameters must be identified [6].

Although psoriasis is a systemic disease that affects quality of life, assessing the inflammatory burden is a challenge. High-sensitivity C-reactive protein (hs-CRP), a commonly used parameter to assess systemic inflammation, is elevated in these patients and correlates with the severity of psoriasis [7]. As an independent risk factor for atherosclerosis, hs-CRP also serves as a useful marker for the assessment of increased cardiovascular risk in psoriasis [4,8].

Uncoupling protein-1 (UCP1), located in the mitochondria, plays a key role in the generation of heat in brown adipose tissue by uncoupling the electron transport chain from ATP production. This unique ability of mammalian brown adipose tissue to convert nutrients into heat helps protect the body from cold and holds promise for combating metabolic diseases in humans. Strategies targeting brown adipose tissue growth and UCP1 activation, such as pharmacological triggering of β3-adrenergic receptors in adipocytes, offer therapeutic approaches to combat diabetes, obesity, and related diseases, even in the absence of natural stimuli [9,10]. UCP1 deficiency is associated with inflammation and endoplasmic reticulum stress [11]. It contributes to the maintenance of cardiovascular health through its antiinflammatory effects and plays an antiatherosclerotic role [12].

Psoriasis, classified as a serious disease by the World Health Organization, has a high prevalence rate and manageable parameters associated with its comorbidities. The development of diagnostic markers could enable earlier diagnosis and treatment, potentially preventing complications associated with the disease. Considering the associations among psoriasis and dyslipidemia, inflammation, disease severity (as reflected by PASI scores), and cardiovascular disease, this study aimed to investigate the alterations in UCP1 level, as an important regulator of lipid metabolism, and to evaluate the potential role of these changes in the pathogenesis of psoriasis.

## 2. Materials and methods

This study involved 30 patients diagnosed with psoriasis from the Dermatology Clinic of the Selçuk University Medical School and 30 healthy individuals who visited our hospital for general health examinations or requested health reports for various reasons to confirm their well-being. The study specifically included patients with PASI scores ranging from 3 to 22, excluding patients with additional conditions besides psoriasis.

After a fasting period of 10–12 h, blood samples were taken from the participants. Routine biochemical tests were performed on these serum samples. Excess serum from these routine tests was divided accordingly. The serum was separated by centrifugation at 3000 rpm for 10 min and part of the serum was then stored at −80 °C until analysis. The study was approved by the Ethics Committee of the Selçuk University Faculty of Medicine (Approval Number 2023/456).

### 2.1. Laboratory measurements

Serum creatinine, triglycerides, total cholesterol, high-density lipoprotein (HDL), low-density lipoprotein (LDL), aspartate aminotransferase (AST), and alanine aminotransferase (ALT) levels were quantified using a Beckman Coulter AU 5800 system (Beckman Coulter, Brea, CA USA). hs-CRP was determined by the immunoturbidimetric method using the Beckman Coulter AU 5800 with measurements expressed in mg/dL.

### 2.2. Measurement of serum UCP1 levels

The UCP1 concentration in serum was determined using a commercial human ELISA kit (Catalog Number E-EL-H1661, Elabscience Biotechnology, Houston, TX, USA) according to the manufacturer’s guidelines. The absorbance in all wells was measured at 450 nm using the CLARIOstar Microplate Reader (BMG LABTECH, Ortenberg, Germany). Serum UCP1 concentrations were derived from a calibration chart prepared using standards and expressed in ng/mL. The inter- and intraassay coefficients of variation for the commercial kit were below 5.88%.

### 2.3. Statistical analysis

Statistical analyses were performed using R Statistical Software Version 4.1.2 (R Foundation for Statistical Computing, Vienna, Austria). The normality of the data was assessed using the Shapiro–Wilk normality test and Q-Q plots, while the Levene test was used to assess variance homogeneity. Numerical variables were expressed as mean ± standard deviation or median with interquartile range (25th percentile–75th percentile) as appropriate. Categorical variables were expressed as number (n) and percentage (%). To determine statistically significant differences or associations between the healthy control group and the psoriasis group in terms of demographic characteristics and biochemical parameters, independent-samples t-tests, Mann–Whitney U tests, and chi-square tests with Yates correction for continuity were performed. In addition, depending on the normality of the data, Pearson and Spearman rho correlation coefficients were evaluated to investigate the relationships between UCP1 levels and other biochemical parameters. Receiver operating characteristic (ROC) curve analysis was performed to determine the diagnostic performance of the UCP1 level to discriminate the psoriasis group from the healthy control group. The area under the curve (AUC) was calculated along with its 95% confidence interval (CI), and the optimal cut-off point was determined using the Youden index. Sensitivity, specificity, negative predictive value (NPV), and positive predictive value (PPV) were then calculated based on this optimal cut-off point. In addition, simple binary logistic regression analysis was conducted to determine the predictive performance of UCP1, categorized as low or high according to the established cut-off point of 7.561 ng/mL (≤7.561 ng/mL defined as a low level of UCP1 and >7.561 ng/mL defined as a high level of UCP1), for psoriatic disease. The odds ratio (OR) was calculated with a 95% CI. A two-sided p-value of less than 5% was considered statistically significant.

## 3. Results

The study comprised 60 participants, including 32 men (53.3%) and 28 women (46.7%), with a mean age of 42.52 ± 12.64 years (range: 19–66 years). Of these participants, 30 were healthy control subjects and the remaining 30 were patients diagnosed with psoriasis.

**Figure 1:**
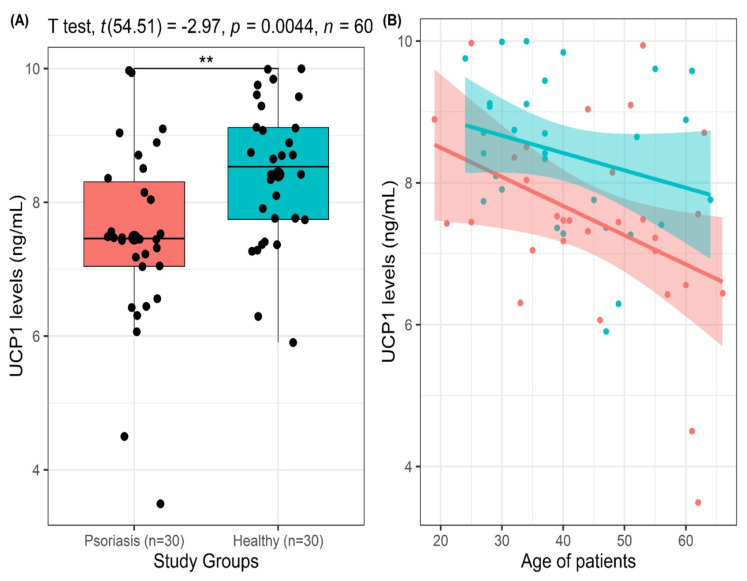
**A)** Box plot showing serum levels of UCP1 in the healthy group and psoriasis group. **B)** Scatter plots showing the relationship between serum UCP1 levels and age in the whole cohort stratified by study group. Pearson r and Spearman rho correlation coefficients are indicated. Red lines are fitted regression lines for each relationship for patients with psoriasis and red shaded areas show the 95% confidence intervals of the regression lines for patients with psoriasis (r = −0.398, p = 0.029). Green lines are fitted regression lines for each relationship for healthy controls and green shaded areas show the 95% confidence intervals of the regression lines for healthy controls (r = −0.268, p = 0.152). Values in black are correlation coefficients for the whole cohort (r = −0.385, p = 0.002).

The demographic characteristics, biochemical parameters, and clinical severity of the psoriasis cases are shown in Table 1. The mean age (44.90 ± 13.35 vs. 40.13 ± 11.61, p = 0.145) and sex distribution (63.3% vs. 43.3% for men, p = 0.196) of the groups were similar. The mean UCP1 level was significantly lower in patients with psoriasis compared to healthy controls (7.47 ± 1.38 vs. 8.42 ± 1.07, p = 0.004; Figure 1A). In terms of biochemical parameters, hs-CRP and triglyceride levels were significantly higher in patients compared to healthy controls, while HDL levels were lower. No significant differences were found in total cholesterol, LDL, creatinine, AST, or ALT between the healthy controls and the patients with psoriasis.

Increased UCP1 values correlated significantly with the age of the participants (Spearman rho = −0.385, p = 0.002; Figure 1B). However, there was no significant correlation between UCP1 level and hs-CRP, triglyceride, total cholesterol, HDL, LDL, creatinine, AST, or ALT levels and PASI scores (Table 2).

**Figure 2:**
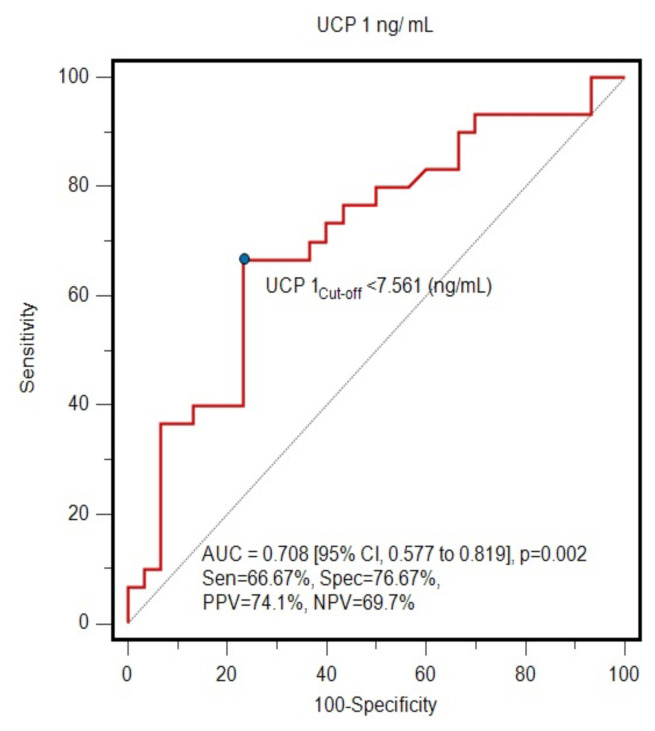
ROC curve analysis of ability of serum UCP1 level to discriminate psoriasis patients from healthy controls. The AUC was 0.708 (95% CI: 0.577–0.819, p = 0.002). Using a cut-off value of 7.561 ng/mL, the sensitivity and specificity were 66.67% (95% CI: 47.2–82.7) and 76.67% (95% CI: 57.7–90.1), respectively.

ROC curve analysis revealed that UCP1 level was a significant marker for the prediction of psoriatic disease (AUC: 0.708, 95% CI: 0.577–0.819, p = 0.002) with specificity of 66.67%, sensitivity of 76.67%, PPV of 69.7%, and NPV of 74.1% (Figure 2). Simple logistic regression analysis showed that an individual with a UCP1 level below 7.561 ng/mL was 73% less likely (OR: 0.27, 95% CI: 0.08–0.94, p = 0.039) to have psoriasis than an individual with a UCP1 level above 7.561 ng/mL.

Since UCP1 could detect patients with psoriasis at an approximate rate of 77% with a cut-off value of >7.561 ng/mL (Figure 2), we consider it a potential biomarker candidate for psoriasis.

## 4. Discussion

Psoriasis is a chronic systemic condition influenced by the immune system, distinguished by the emergence of red, hardened, flaky, itchy, and frequently discomforting patches on the skin [13]. It is associated with a higher likelihood of major adverse cardiac events, cardiovascular death, and various tissue and organ diseases. The severity of psoriatic skin issues correlates with both systemic inflammation and the scope of cardiovascular disease [14]. Inflammation is a pivotal link between psoriasis and atherosclerosis, and several lines of evidence indicate that psoriasis is associated with enhanced atherosclerosis and an increased risk of cardiovascular disease [15]. Moreover, psoriasis is acknowledged as an immune-mediated inflammatory condition that affects the entire system rather than being solely confined to the skin. Its presence is linked to metabolic disruptions resulting from persistent inflammation [16]. The higher occurrence of cardiometabolic conditions in psoriasis stems from both pervasive systemic inflammation and a heightened prevalence of conventional risk factors for cardiometabolic diseases [17]. Conventional systemic inflammation markers like hs-CRP are partially associated with the severity of psoriasis but do not provide sufficient information about the extent of the disease involvement. This situation underscores the importance of the need for new biomarkers to understand the impact of psoriasis on systemic inflammation and comorbidities [18]. Psoriasis is also associated with an array of comorbidities, including hypertension, glucose intolerance, obesity, dyslipidemia, and cardiovascular events [19].

UCP1 is primarily found in brown adipose tissue and is of crucial importance for releasing heat by dissipating the proton gradient, effectively separating respiration from ATP synthesis [20]. Its deficiency leads to several disorders including obesity, diabetes mellitus, atherosclerosis, and more. UCP1 deficiency is associated with inflammation and endoplasmic reticulum stress. Additionally, it aids in maintaining cardiovascular health through its antiinflammatory effects and plays an antiatherosclerotic role [12]. In our study, we found that UCP1 levels in psoriasis patients (7.47 ± 1.38 ng/mL) were lower compared to the control group (8.42 ± 1.07 ng/mL). It is generally recognized in the literature that psoriasis is the dermatological disease most associated with lipid metabolism [21]. Therefore, to evaluate the impact of psoriasis on lipid metabolism and its relationship with systemic inflammation, only psoriasis patients were included as a patient group in this study and compared with a control group of healthy individuals. This approach allowed us to conduct a specific assessment to better understand the role of lipid metabolism in the pathogenesis of psoriasis. The effects of other inflammatory dermatological diseases on UCP1 levels are considered a separate research topic that could be studied in comparison to psoriasis. As the primary aim of this study was to determine the role of UCP1 in psoriasis patients, other inflammatory skin diseases were not considered.

Statistically significant differences were observed in this study between the patient and control groups for triglycerides, hs-CRP, and HDL (p < 0.05). Research has shown that patients with psoriasis exhibit alterations in lipid metabolism, including changes in total cholesterol, LDL, triglycerides, and HDL [22–24]. These lipid abnormalities are significant as they contribute to the development of atherosclerosis, a process characterized by the accumulation of cholesterol and inflammatory cells in the arterial walls [25,26]. In our study, the patient group exhibited notably elevated triglyceride levels in contrast to the control group, confirming these findings. There were statistically noteworthy variances observed between the patient and control groups concerning triglycerides, hs-CRP, UCP1, and HDL (p < 0.05), as illustrated in Table 1. These lipid alterations suggest a potential association between psoriasis and dyslipidemia, which is a known risk factor for cardiovascular diseases [27–29]. Moreover, psoriasis has been associated with systemic inflammation and decreased HDL levels, both significant elements in the emergence of cardiovascular conditions [30]. The association between psoriasis and dyslipidemia has also been supported by studies demonstrating elevated serum total cholesterol, LDL, and triglyceride levels, as well as lower serum HDL levels in psoriasis patients [31]. Additionally, psoriasis has been found to be accompanied by significant increases in total cholesterol, LDL, and very low-density lipoprotein, further indicating a potential link between psoriasis and dyslipidemia [24]. UCP1 plays a crucial role in lipid metabolism and energy expenditure [32]. The absence of UCP1 boosts the production of monounsaturated fatty acids in adipose tissue and their transportation to the liver, highlighting its impact on regulating lipid metabolism [33]. UCP1 also plays a role in regulating lipid metabolism in white adipose tissue and increasing energy consumption [34]. Assessing oxidative stress and inflammatory markers in individuals with psoriasis can unveil their influence on the onset and advancement of additional health issues. This underscores the crucial role of timely prevention measures in enhancing quality of life for those with psoriasis [35]. As shown in Table 2, correlation analysis also revealed a negative correlation between UCP1 and age. This result is consistent with data in the literature and indicates that UCP1 activity in the inner mitochondrial membrane of brown adipocytes decreases significantly with increasing age [36,37]. It is suspected that reduced UCP1 levels play important roles in the development of age-related type 2 diabetes, obesity, and various other diseases [38,39] and that the protective effect of UCP1 decreases with age. Therefore, treatment approaches for increasing UCP1 levels could be considered as a possible option.

Due to its ability to identify 66.67% of psoriasis patients at levels above 7.561 ng/mL and to differentiate 76.67% of healthy individuals at levels below that value, UCP1 can be considered as a potential marker in psoriasis. These values of sensitivity and specificity may be limited for diagnostic or follow-up purposes. However, this is the first study to investigate the association between UCP1 and psoriasis, and the results provide important information for the literature. Further studies including other inflammatory skin diseases are needed to confirm and extend these findings.

While patients with a history of inflammatory disease and those using antiinflammatory drugs were excluded from the study, complete standardization across groups could not be achieved due to factors such as age, sex, diet, or others. Therefore, the changes in these biomarkers might be influenced by reasons beyond our control and this constitutes a limitation of the present study. Additionally, the lack of vascular assessments (carotid intima–media thickness, arterial stiffness, etc.) of the patients and the lack of analysis regarding subcutaneous fat tissue are limitations of this study.

## Figures and Tables

**Table 1:** Demographic characteristics, biochemical parameters, and clinical severity scores of the participants in the control group and psoriasis group.

	Control (n = 30)	Psoriasis (n = 30)	p
Demographic characteristics			
Age, years	40.13 ± 11.61	44.90 ± 13.35	0.145^1^
Sex, male/female	13 (43.3)/17 (56.7)	19 (63.3)/11 (36.7)	0.196^2^
Biochemical parameters			
UCP1, ng/mL	8.42 ± 1.07	7.47 ± 1.38	0.004^1^
hs-CRP, mg/dL	1.26 [0.95–1.93]	2.24 [1.09–3.67]	0.019^3^
Triglycerides, mg/dL	90.5 [67.25–109.75]	133.5 [87.25–204.25]	0.011^3^
Total cholesterol, mg/dL	183 ± 33.16	176.8 ± 30.5	0.454^1^
HDL, mg/dL	47.73 ± 10.62	38.50 ± 8.73	<0.001^1^
LDL, mg/dL	108.37 ± 26.34	113.43 ± 31.29	0.500^1^
Creatinine, mmol/L	0.80 ± 0.16	0.74 ± 0.15	0.175^1^
AST, U/L	20.23 ± 4.42	19.67 ± 5.57	0.664^1^
ALT, U/L	14.5 [9.25–18]	14 [11–22.75]	0.464^3^
Clinical severity score:			
Psoriasis Area and Severity Index		8.28 ± 4.17 [3–22]	

Data are presented as mean ± standard deviation, median [interquartile range], or number and (%).

1 Independent-samples t-test;

2 chi-square test with Yates continuity correction;

3 Mann–Whitney U test.

**Table 2:** Relationships between UCP1 levels and demographic characteristics, biochemical parameters, and clinical severity scores for all participants.

	UCP1 (ng/mL)	

	Correlation coefficient	p
Demographic characteristics		
Age, years	Pearson r = −0.385	0.002
Biochemical parameters		
hs-CRP, mg/dL	Spearman rho = −0.129	0.326
Triglycerides, mg/dL	Spearman rho = 0.055	0.679
Total cholesterol, mg/dL	Pearson r = 0.012	0.929
HDL, mg/dL	Pearson r = −0.095	0.469
LDL, mg/dL	Pearson r = −0.018	0.894
Creatinine, mmol/L	Pearson r = 0.132	0.313
AST, U/L	Pearson r = −0.002	0.988
ALT, U/L	Spearman rho = −0.109	0.406
Clinical severity score:		
Psoriasis Area and Severity Index	Spearman rho = −0.251	0.180
